# Mutation Spectrum Induced by 8-Bromoguanine, a Base Damaged by Reactive Brominating Species, in Human Cells

**DOI:** 10.1155/2017/7308501

**Published:** 2017-09-30

**Authors:** Kazuya Shinmura, Hisami Kato, Masanori Goto, Hong Tao, Yusuke Inoue, Satoki Nakamura, Haruki Yoshida, Emi Tsuzaki, Haruhiko Sugimura

**Affiliations:** ^1^Department of Tumor Pathology, Hamamatsu University School of Medicine, Hamamatsu 431-3192, Japan; ^2^Division of Tumor Pathology, Department of Pathology, Asahikawa Medical University, Asahikawa 078-8510, Japan

## Abstract

To date, the types of mutations caused by 8-bromoguanine (8BrG), a major base lesion induced by reactive brominating species during inflammation, in human cells and the 8BrG repair system remain largely unknown. In this study, we performed a *supF* forward mutation assay using a shuttle vector plasmid containing a single 8BrG in three kinds of human cell lines and revealed that 8BrG in DNA predominantly induces a G → T mutation but can also induce G → C, G → A, and delG mutations in human cells. Next, we tested whether eight kinds of DNA glycosylases (MUTYH, MPG, NEIL1, OGG1, SMUG1, TDG, UNG2, and NTHL1) are capable of repairing 8BrG mispairs with any of the four bases using a DNA cleavage activity assay. We found that both the SMUG1 protein and the TDG protein exhibit DNA glycosylase activity against thymine mispaired with 8BrG and that the MUTYH protein exhibits DNA glycosylase activity against adenine mispaired with 8BrG. These results suggest that 8BrG induces some types of mutations, chiefly a G → T mutation, in human cells, and some DNA glycosylases are involved in the repair of 8BrG.

## 1. Introduction

The fact that inflammation is a major cancer predisposition factor is supported by multiple lines of evidence [1, 2]. At inflammatory sites, in the presence of plasma halides, the enzymes eosinophil peroxidase and myeloperoxidase, which are released by eosinophils and neutrophils, respectively, generate hypobromous acid, a reactive brominating species [3, 4]. Hypobromous acid works as a potent oxidant that oxidizes the cellular material of invading pathogens under inflammatory conditions; however, excess amounts of hypobromous acid can also damage host DNA, proteins, and lipids [5, 6]. 8-Bromoguanine (8BrG) is one type of damaged base lesions that is known to be induced by hypobromous acid [5]. 8BrG has actually been detected in human liver and urine [7]. Interestingly, the significant elevation of 8BrG levels, as quantified using liquid chromatography-tandem mass spectrometry, has been reported in urine samples from patients with diabetes mellitus [7], a disease considered to be associated with oxidative stress and inflammation [8].

Since the presence of oxidatively damaged bases, such as 8-oxoguanine (8oxoG), in DNA can cause mutation [9–12], it is possible that the generation of 8BrG at sites of inflammation could lead to mutation. In this regard, a previous *in vitro* study of translesion synthesis catalyzed by Pol*α*, Pol*κ*, and Pol*η* using 8BrG-containing oligonucleotides reported that Pol*α* induced a one-base deletion at a low frequency and that Pol*κ* promoted a one-base deletion and the misincorporation of G, A, and T opposite the lesion at a relatively low frequency, whereas Pol*η* bypassed 8BrG in an error-free manner [13]. These findings are in contrast to the fact that no miscoding events were observed in the cases of 8-bromoadenine and 5-bromocytosine, the other lesions induced by hypobromous acid [13]. Another study reported that both Pol*α* and Pol*β* not only incorporated C (a correct base) opposite the 8BrG lesion but also led to a one-base deletion or the incorporation of A (an incorrect base) at a low frequency [14]. Thus, the results of these two studies indicated that the miscoding properties of 8BrG lesions vary depending on the DNA polymerase. Additionally, other human DNA polymerases for which the miscoding properties of 8BrG lesions have not been reported do exist [15]; therefore, the types of mutations that are most predominantly induced by 8BrG in human cells remain uncertain.

Moreover, since the DNA glycosylase proteins OGG1 and MUTYH are involved in the repair of 8oxoG [16, 17], any of several DNA glycosylases could be involved in the repair of 8BrG. DNA glycosylases are members of proteins involved in base excision repair, and they catalyze the first step of this repair process by eliminating the mispaired bases by cleaving the *N*-glycosidic bond. Regarding the repair of 8oxoG, OGG1 excises 8oxoG mispaired with C, whereas MUTYH excises A mispaired with 8oxoG [16, 17]. As for other damaged bases, uracil (U) is a substrate of the DNA glycosylases UNG and SMUG1, thymine glycol is a substrate of NTHL1 and NEIL1, and 3-methyladenine and 1,*N*^6^-ethenoadenine are the substrates of MPG [18]. Even if the bases are undamaged bases, a mismatch can be repaired by DNA glycosylases; for example, undamaged T mispaired with G is a substrate of TDG [19]. These combinations of DNA substrates and DNA glycosylase are representative only, and DNA glycosylases actually possess repair activities towards larger numbers of damaged bases. Since DNA mispairs arising from the existence of damaged bases can cause mutations [20, 21], an understanding of the repair system for damaged bases by DNA glycosylases is important. At present, however, the human DNA glycosylases involved in 8BrG repair have not yet been revealed. Since cancer is caused by certain genetic changes, including DNA mutations, that control the way human cells function adequately, especially how they grow and divide, an understanding of the system responsible for mutations and a means of avoiding mutation are important. Therefore, in this study, we investigated what kinds of mutations are induced by 8BrG in human cells and whether any DNA glycosylases are involved in 8BrG repair.

## 2. Materials and Methods

### 2.1. Cell Cultures

The human lung cancer cell line H1299 and the human glioblastoma cell line LN428 were obtained from the American Type Culture Collection (Manassas, VA, USA) and Trevigen (Gaithersburg, MD, USA), respectively. The 16HBE14o- cell line (Simian virus 40-transformed human bronchial epithelial cells) [22] was a gift from Dr. D.C. Gruenert (California Pacific Medical Center Research Institute, San Francisco, CA, USA) via Dr. T. Kaneko (Department of Internal Medicine, Yokohama City University, School of Medicine, Yokohama, Japan). The cells were maintained at 37°C in RPMI 1640 medium supplemented with 10% fetal bovine serum and penicillin/streptomycin under a 5% CO_2_ atmosphere.

### 2.2. Construction of a Shuttle Vector Plasmid Containing an 8BrG Residue

A shuttle vector pMY189, which contains the bacterial suppressor tRNA (*supF*) gene [23], was used for the construction of pMY189 containing a single 8BrG:cytosine pair. First, *E. coli* XL1-Blue MRF' (Stratagene, La Jolla, CA, USA) and R408 Helper Phage (Stratagene) were used to prepare single-stranded pMY189 DNA, and 30 *μ*g of the single-stranded plasmid pMY189 and a 5-fold molar excess of 5′-phosphorylated 24-mer oligonucleotide with a single 8BrG at nucleotide position 159 of the *supF* gene [5′-CGA CTT CGA A(8BrG)G TTC GAA TCC TTC-3′] (Japan Bio Services, Saitama, Japan) were annealed in a reaction mixture. Forty units of T4 DNA polymerase (Takara, Kyoto, Japan), 600 *μ*M of deoxynucleotide triphosphate, 2400 units of T4 DNA ligase (New England Biolabs, Beverly, MA, USA), and 1 mM of ATP were added to the reaction mixture, and the mixture was incubated at 37°C for 4 h. Then, closed circular pMY189 containing an 8BrG was isolated using cesium chloride-ethidium bromide density gradient centrifugation. To prepare wild-type pMY189, an oligonucleotide without modified bases [5′-CGA CTT CGA AGG TTC GAA TCC TTC-3′] was used, and a closed circular wild-type pMY189 was obtained in the same manner.

### 2.3. *supF* Forward Mutation Assay

A *supF* forward mutation assay was performed as described previously [24] with some modifications. Cells were transfected with the shuttle plasmid wild-type pMY189 or 8BrG-containing pMY189 using Lipofectamine 2000 reagent (Invitrogen, Carlsbad, CA, USA). After 48 h, the propagated plasmids were extracted from the cells using a QIAprep Spin Miniprep Kit (Qiagen, Valencia, CA, USA) and digested with *Dpn*I restriction enzyme to eliminate unreplicated plasmids with the bacterial methylation pattern. After purification with Amicon Ultra Centrifugal Filter Units (Millipore, Bedford, MA, USA), the plasmids were introduced into the KS40/pKY241 indicator *E. coli* strain [25] using electroporation. The transformants were plated onto LB agar plates containing 50 *μ*g/mL of nalidixic acid, 150 *μ*g/mL of ampicillin, and 30 *μ*g/mL of chloramphenicol, together with isopropyl-*β*-D-thiogalactopyranoside (IPTG) and 5-bromo-4-chloro-3-indolyl-*β*-D-galactopyranoside (X-gal). White colonies on this plate were counted as *supF* mutants. The mutation frequencies were calculated as the number of *supF* mutants per the total number of transformants, which were counted on LB plates containing ampicillin, chloramphenicol, IPTG, and X-gal. The mutations in the *supF* gene were then analyzed using polymerase chain reaction (PCR) with a set of primers (5′-TGT AAA ACG ACG GCC AGT-3′ and 5′-ATC TCA AGA AGA TCC TTT GAT C-3′) and a subsequent sequencing analysis as described previously [26]. The numbers of mutant colonies analyzed using PCR and sequencing are summarized in Supplementary Table S1 available online at https://doi.org/10.1155/2017/7308501.

### 2.4. Preparation of Recombinant Proteins

Recombinant DNA glycosylase proteins were expressed and purified as described previously [27–29]. The reference numbers for MUTYH, MPG, NEIL1, OGG1, SMUG1, TDG, UNG2, and NTHL1 proteins are NP_001041639.1, NP_002425.2, NP_078884.2, NP_002533.1, NP_001230716.1, NP_003202.3, NP_550433.1, and NP_002519.1, respectively. Briefly, MUTYH, MPG, NEIL1, UNG2, and NTHL1 proteins fused with the His_6_ tag were expressed in a pET system using a pET25b(+) expression vector (Novagen, Madison, WI, USA) and *E. coli* BL21-CodonPlus(DE3)-RP-competent cells (Stratagene) and then purified using TALON metal affinity resins (Clontech, Palo Alto, CA, USA). OGG1, SMUG1, and TDG proteins fused with the GST tag were expressed using a pGEX-1*λ*T or pGEX-2T expression vector (Amersham Biosciences, Piscataway, NJ, USA) and *E. coli* BL21 (Stratagene) and then purified with glutathione Sepharose 4B or glutathione Sepharose 4 Fast Flow (Amersham Biosciences). The qualities and concentrations of the proteins were determined by resolving the proteins with SDS-polyacrylamide gel electrophoresis (PAGE) and staining them with Coomassie Brilliant Blue; ImageJ software (National Institutes of Health, Bethesda, MD, USA) was then used for quantification.

### 2.5. DNA Cleavage Activity Assay

A 30-mer oligonucleotide containing a single 8BrG (5′-CTG GTG GCC TGA C[8BrG]C ATT CCC CAA CTA GTG-3′) (Japan Bio Services) was ^32^P-labeled at the 5′ terminus with a MEGALABEL kit (Takara) and [*γ*-^32^P]ATP (PerkinElmer, Tokyo, Japan) and then annealed to a complementary oligonucleotide containing an undamaged T, C, G, or A opposite the 8BrG. We also prepared a double-stranded oligonucleotide in which the 5′ terminus of oligonucleotides containing an unmodified base opposite the 8BrG was ^32^P-labeled. The labeled oligonucleotide (2.5 nM) and 300 fmoles of each DNA glycosylase protein were incubated in 20 *μ*L of the reaction mixture, which was described previously [29], at 37°C for the indicated time, and the mixture was then treated with 0.1 N NaOH. After the alkali treatment, the mixture was denatured and subjected to 20% PAGE. A ^32^P-labeled marker oligonucleotide was used as a size marker for the cleavage products. The radioactivities of the intact and cleaved oligonucleotides were quantified using an FLA-3000 fluoro image analyzer (Fuji Film, Tokyo, Japan) and Image Gauge software (Fuji Film). For a kinetic study of DNA cleavage, TDG proteins were reacted with various amounts (1.25, 2.5, 5, 10, 20, and 40 nM) of the T:8BrG substrate at 37°C for 3 min, whereas SMUG1 proteins were reacted with various amounts (2.5, 5, 10, and 20 nM) of the T:8BrG substrate at 37°C for 15 min. Lineweaver-Burk plots representing the reciprocal of the initial rates of thymine excision versus the reciprocal of the substrate concentrations were utilized to determine the Michaelis constant (*K*m) and the catalytic constant (*K*cat).

### 2.6. Sequencing Analysis

Genomic DNA was extracted from LN428 cells, and all the coding exons of the *TDG* and *SMUG1* genes and their boundary regions were amplified using PCR with HotStarTaq DNA polymerase (Qiagen). The PCR primer sequences are summarized in Supplementary Table S2. The PCR-amplified products were directly sequenced using a BigDye Terminator Cycle Sequencing Reaction Kit (Applied Biosystems, Tokyo, Japan) and an ABI 3130 Genetic Analyzer (Applied Biosystems).

### 2.7. Establishment of Stable Inducible Cell Lines

H1299 cells were transfected with a PiggyBac cumate switch inducible vector (System Biosciences, Mountain View, CA, USA) for the expression of MUTYH together with the PiggyBac transposase vector (System Biosciences). Positively transposed cells were then selected using puromycin (1.2 *μ*g/mL: Clontech). We also prepared cells transfected with an empty (parental) PiggyBac cumate switch inducible vector and transposase vector.

### 2.8. Western Blot Analysis

Cultured cells were lysed in a buffer containing 50 mM HEPES-KOH (pH 7.5), 150 mM NaCl, 0.1% sodium dodecyl sulfate, 1% Triton X-100, 0.5% sodium deoxycholate, 100 mM sodium fluoride, 1 mM sodium orthovanadate, and protease inhibitor cocktail (Sigma-Aldrich, St. Louis, MO, USA). A Western blot analysis was performed using an anti-MUTYH monoclonal antibody (clone 4D10; Abnova, Taipei, Taiwan) or an anti-GAPDH monoclonal antibody (clone 6C5; Abcam, Cambridge, UK). Immunoreactivity was visualized using an ECL chemiluminescence system (GE Healthcare Bio-Science, Piscataway, NJ, USA).

### 2.9. Statistical Analysis

The statistical analysis was performed using an unpaired *t*-test and JMP version 9.0 software (SAS Institute, Cary, NC, USA). *P* values of less than 0.05 were considered statistically significant.

## 3. Results

### 3.1. Induction of Mutations by 8BrG in Human Cells

**Figure 1:**
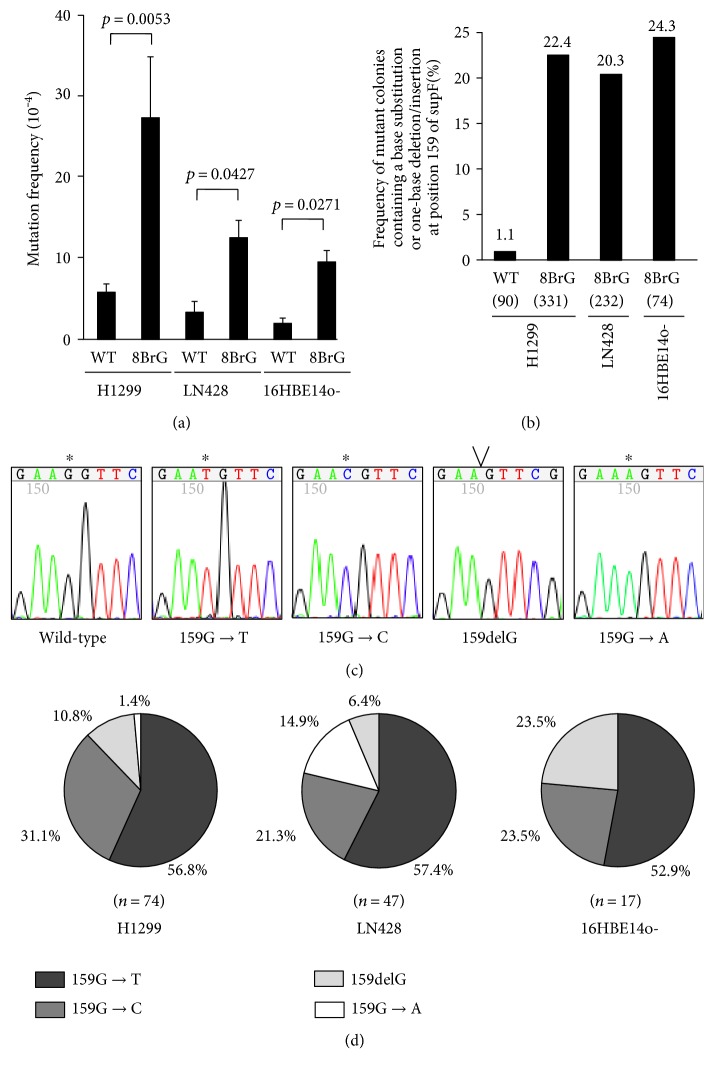
Induction of mutations by 8-bromoguanine (8BrG) in human cells. (a) Comparison of the mutation frequency of the *supF* gene in the pMY189 plasmid using a *supF* forward mutation assay in the human H1299, LN428, and 16HBE14o- cell lines. The mutation frequency was compared between wild-type pMY189 and 8BrG-containing pMY189, which has an 8BrG residue at position 159 of *supF*. The data are shown as the means ± standard error. (b) Frequency of mutant colonies containing a base substitution mutation or one-base deletion mutation at position 159 of *supF* on wild-type pMY189 and/or 8BrG-containing pMY189 in a *supF* forward mutation assay in H1299, LN428, and 16HBE14o- cells. The total number of mutant colonies that were analyzed is shown in parentheses. (c) Representative results of *supF* mutations in 8BrG-containing pMY189 plasmids replicated in H1299 cells. Sequencing electropherograms show a G → T, G → C, delG, or G → A mutation at position 159 of the *supF.* A mutated site and a deleted site were marked by an asterisk and “V,” respectively. The leftmost is the wild-type *supF* sequence. (d) Proportion of mutation types detected at position 159 of *supF* on 8BrG-containing pMY189 plasmids replicated in H1299, LN428, and 16HBE14o- cells. The total number of mutations at position 159 of *supF* is shown in parentheses.

To elucidate the mutagenicity of 8BrG in human cells, a *supF* forward mutation assay with a shuttle plasmid, pMY189, was performed in two human tumor cell lines (H1299 and LN428) and a human normal cell line (16HBE14o-). We constructed a pMY189 plasmid containing a single 8BrG residue at position 159 of the *supF* gene, and we compared the mutation frequency between the cells transfected with wild-type pMY189 and that of those transfected with 8BrG-containing pMY189. As a result, the mutation frequency of *supF* in the H1299, LN428, and 16HBE14o- cells was significantly increased by the introduction of 8BrG (*P* = 0.0053, *P* = 0.0427, and *P* = 0.0271, resp.) (Figure 1(a)). We further investigated the type of mutation contained in the *supF* mutant colony using PCR and a subsequent sequencing analysis of the *supF* region and found that the percentage of mutant colonies containing a base substitution or one-base insertion/deletion at position 159 of *supF* was markedly higher in the 8BrG-containing pMY189 (22.4%) than in the wild-type pMY189 (1.1%) in H1299 cells (Figures 1(b) and 1(c)). The frequency was also high in the 8BrG-containing pMY189 replicated in LN428 cells (20.3%) and in 16HBE14o- cells (24.3%) (Figure 1(b)). Among the types of mutations at position 159 of *supF*, a G → T mutation was the most frequent in H1299 (56.8%, 42/74), LN428 (57.4%, 27/47), and 16HBE14o- (52.9%, 9/17) cells, while G → C (21.3%-31.1%), G → A (0%-14.9%), and delG (6.4%-23.5%) mutations were also found at lower frequencies (Figure 1(d)). A similar result was obtained when a pMY189 plasmid containing an 8BrG residue at position 144 of the *supF* gene was used for a *supF* forward mutation assay in H1299 cells (Supplementary Figure S1). These results suggested that 8BrG induces the G → T mutation most frequently but it also induces other types of mutations, such as G → C, G → A, and delG, in human cells.

### 3.2. Involvement of DNA Glycosylase Proteins in 8BrG Repair

**Figure 2:**
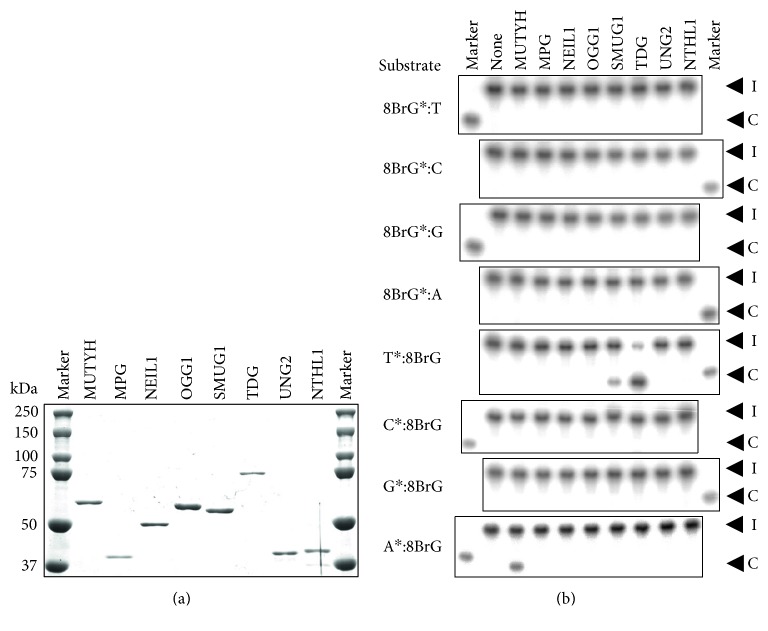
Evaluation of repair activities of eight DNA glycosylase proteins against the 8-bromoguanine- (8BrG-) containing double-stranded oligonucleotides. (a) Expression and purification of the DNA glycosylase proteins MUTYH, MPG, NEIL1, OGG1, SMUG1, TDG, UNG2, and NTHL1. The proteins were resolved using SDS-PAGE and stained with Coomassie Brilliant Blue. (b) The abilities of the DNA glycosylase proteins MUTYH, MPG, NEIL1, OGG1, SMUG1, TDG, UNG2, and NTHL1 to repair eight kinds of 30-mer double-stranded oligonucleotides containing 8BrG were examined using a DNA cleavage activity assay. Each DNA glycosylase protein was allowed to act on double-stranded oligonucleotides containing 8BrG paired with each unmodified base or containing each unmodified base paired with 8BrG at 37°C for 60 min. The asterisks show the 5′-^32^P-labeled oligonucleotides. A ^32^P-labeled marker oligonucleotide was used as a size marker for the cleavage products. The intact oligonucleotides and cleavage products are indicated by “I” and “C,” respectively.

Next, we attempted to investigate whether DNA glycosylase proteins are involved in the repair of 8BrG. First, eight kinds of DNA glycosylase proteins (MUTYH, MPG, NEIL1, OGG1, SMUG1, TDG, UNG2, and NTHL1) were expressed and purified (Figure 2(a)). To confirm that these DNA glycosylase proteins prepared for our assay possessed enzymatic activity, we examined the repair activity of each protein towards an oligonucleotide containing a previously known substrate [16–19, 27] using a DNA cleavage assay. Substantial repair activities were observed for all the proteins (Supplementary Figure S2). Double-stranded oligonucleotides containing 8BrG paired with unmodified T, C, G, or A were also prepared as substrates. For each double-stranded oligonucleotide, ^32^P-labeling at the 5′ terminus was performed for 8BrG-containing oligonucleotide or unmodified oligonucleotides paired with 8BrG (Supplementary Figure S3), meaning that we were able to evaluate DNA glycosylase activity towards the 8BrG itself and each unmodified base opposite 8BrG. Next, eight kinds of DNA glycosylase proteins were reacted with eight kinds of oligonucleotide substrates, and the reaction mixtures were then subjected to PAGE after alkali treatment. As a result, none of the DNA glycosylases showed cleavage activity towards 8BrG paired with T, C, G, or A or towards C or G paired with 8BrG (Figure 2(b)). However, when the cleavage activity against unmodified T paired with 8BrG was examined, SMUG1 and TDG proteins, but not the six other DNA glycosylases that were examined, showed cleavage activity (Figure 2(b)). In addition, when the cleavage activity against unmodified A paired with 8BrG was examined, MUTYH protein, but not the seven other DNA glycosylases that were examined, showed cleavage activity (Figure 2(b)).

### 3.3. Involvement of SMUG1 and TDG Proteins in 8BrG Repair

**Figure 3:**
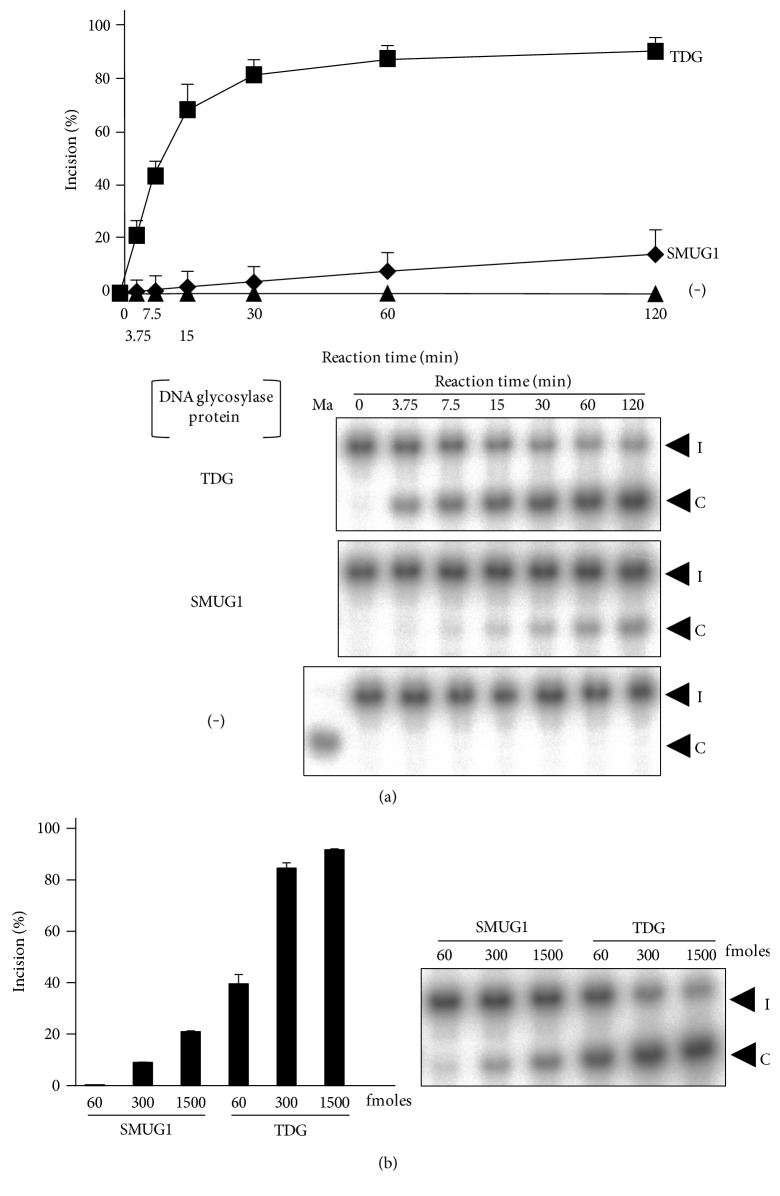
Excision of thymine mispaired with 8-bromoguanine (8BrG) by SMUG1 and TDG proteins. (a) Time-course assay for the cleavage of 30-mer double-stranded oligonucleotides containing a T:8BrG mispair by SMUG1 and TDG proteins. Each protein or no DNA glycosylase proteins (−) were incubated at 37°C for 0–120 min with a T:8BrG-containing oligonucleotide. The amount of cleavage products as a proportion of the total oligonucleotides was calculated as the % incision. The % incision values were shown as the means ± standard deviations of data from three independent experiments. The lower panels show representative results of the DNA cleavage activity assays. (b) Protein concentration dependency of cleavage of double-stranded oligonucleotide containing a T:8BrG mispair by SMUG1 and TDG proteins. Each protein was incubated at 37°C for 60 min with a T:8BrG-containing oligonucleotide. The amount of cleavage products as a proportion of the total oligonucleotides was calculated as the % incision. Data are shown as the means ± standard deviations. The right panel shows a representative result. “I” and “C” indicate intact oligonucleotides and cleaved oligonucleotides, respectively (a and b).

Next, to further investigate the cleavage activity of SMUG1 and TDG, both proteins were reacted with T:8BrG substrate for various time periods (i.e., a time-course assay) and the percentage of cleaved products per total oligonucleotide was calculated and expressed as the percentage incision. The time-course assay demonstrated that both SMUG1 and TDG cleaved the T:8BrG substrate (Figure 3(a)). As a positive control, the cleavage activity of TDG against a T:G substrate [19] was also observed (Supplementary Figure S4). In addition, the excision statuses of SMUG1 and TDG proteins for T paired with G, C, or A, instead of 8BrG, were determined (Supplementary Figure S5). Moreover, when various amounts of reacted protein were used in the DNA cleavage activity assay, an increase in the protein amount led to an increase in the percentage incision for both the SMUG1 and TDG proteins (Figure 3(b)). To further investigate the activities of SMUG1 and TDG proteins, the kinetic parameters of the glycosylase reaction for T:8BrG mispair by these proteins were determined (Table 1). The *K*cat/*K*m value, representing the catalytic efficiency, of the TDG protein was higher than that of the SMUG1 protein, consistent with the results shown in Figure 3(). Finally, since the percentage of 159G → A mutation was higher for LN428 cells than for 16HBE14o- cells (Figure 1(d)), we examined whether nucleotide mutations of the *SMUG1* and *TDG* genes are present in LN428 cells and whether the SMUG1 and TDG expression levels are reduced in LN428 cells. A Sanger sequencing analysis revealed no somatic mutations in any of the coding exons of the *SMUG1* and *TDG* genes in LN428 cells, while the expression level of TDG protein, but not of SMUG1 protein, was mildly reduced in LN428 cells, compared with 16HBE14o- cells (Supplementary Figure S6), which might be related to the results shown in Figure 1(d). All above results suggested that both the SMUG1 and TDG proteins exhibit DNA glycosylase activity towards DNA containing a T:8BrG mispair.

### 3.4. Involvement of MUTYH Protein in 8BrG Repair

**Figure 4:**
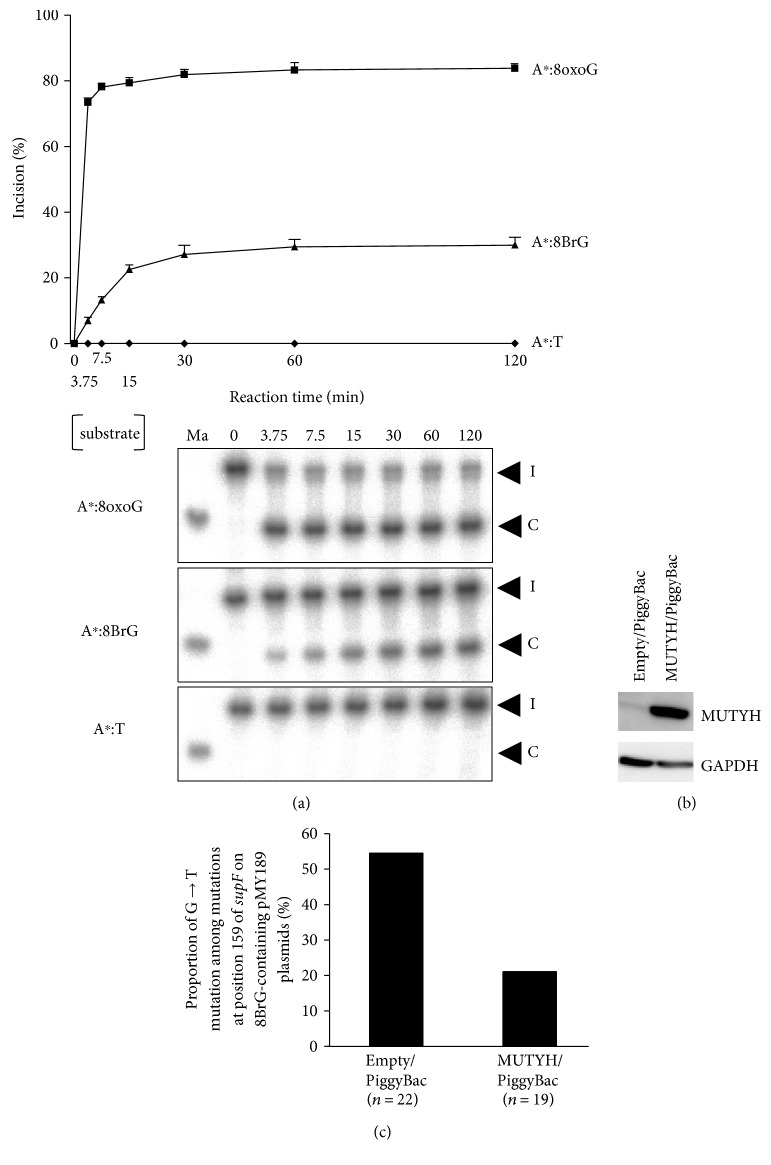
Excision of adenine mispaired with 8-bromoguanine (8BrG) by MUTYH protein. (a) Time-course assay for the cleavage of 30-mer double-stranded oligonucleotides containing an A:8BrG or an A:8oxoG mispair or not containing a mispair (A:T) by MUTYH protein. The protein was incubated at 37°C for 0–120 min with each oligonucleotide. The amount of cleavage products as a proportion of the total oligonucleotides was calculated as the % incision. The % incision values were shown as the mean ± standard deviation of data from three independent experiments. The lower panels show representative results of the DNA cleavage activity assays. The asterisks show the 5′-^32^P-labeled oligonucleotides. (b) Detection of MUTYH proteins in cumate-inducible stable H1299 lung cancer cell lines designed to express MUTYH in the presence of cumate; the MUTYH proteins were detected using a Western blot analysis. Empty vector-transposed cells were used as a control. (c) Proportion of G → T mutation among mutations at position 159 of *supF* on 8BrG-containing pMY189 plasmids replicated in MUTYH-overexpressing H1299 cells and empty vector-transposed H1299 cells. The total number of mutations at position 159 of *supF* is shown in parentheses.

Next, to investigate the cleavage activity of MUTYH further, the percentage incision of A:8BrG substrate was measured for MUTYH protein in a time-course assay. In this analysis, an A:8oxoG substrate was used as a positive control [10, 17]. The time-course assay demonstrated that MUTYH possessed cleavage activity towards both A:8oxoG and A:8BrG substrates and that the A:8oxoG substrate was more efficiently cleaved by MUTYH than the A:8BrG substrate (Figure 4(a)). Since the activity of MUTYH towards A:8BrG is hypothesized to lead to the suppression of G → T mutations and MUTYH is known to be capable of suppressing G → T mutations caused by 8oxoG in human cell lines [26], we examined whether MUTYH is capable of suppressing G → T mutations caused by 8BrG. We established human H1299 lung cancer cells capable of inducibly expressing MUTYH using the PiggyBac transposon vector system (Figure 4(b)) and performed a *supF* forward mutation assay using a pMY189 plasmid containing an 8BrG at position 159 of the *supF* gene in the established cell lines. The results showed that the proportion of G → T mutation among mutations at position 159 of *supF* was markedly lower in MUTYH-overexpressing H1299 cells (21.1%, 4/19) than in empty vector-transposed H1299 cells (54.5%, 12/22) (Figure 4(c)), suggesting that MUTYH possesses the ability to suppress G → T mutations caused by 8BrG. These results suggested that MUTYH protein exhibits DNA glycosylase activity towards DNA containing an A:8BrG mispair.

## 4. Discussion

To date, the type of mutations caused by 8BrG in human cells and the 8BrG repair system remain largely unknown. In this study, we performed a *supF* forward mutation assay using a shuttle vector plasmid containing a single 8BrG residue in three kinds of human cells and revealed that 8BrG in DNA predominantly induces a G → T mutation but can also induce G → C, G → A, and delG mutations in human cells. We also performed a DNA cleavage activity assay examining 8BrG-containing double-stranded oligonucleotides and discovered that both SMUG1 and TDG proteins are capable of excising T mispaired with 8BrG in DNA and that MUTYH protein is capable of excising A mispaired with 8BrG in DNA. Thus, our results suggest that 8BrG is mutagenic and that some DNA glycosylases are involved in the repair of 8BrG, providing a new and important link between 8BrG generation at sites of inflammation and cancer.

In the present study, the types of mutation caused by 8BrG were revealed in human cells for the first time. In this regard, the results of previous studies by Sassa et al. [13] and Efrati et al. [14] indicated that the miscoding properties of 8BrG lesions vary depending on the DNA polymerase and implied that 8BrG can cause delG, G → C, G → T, and G → A mutations. Although other human DNA polymerases do exist [15] and although the effects of these DNA polymerases on the miscoding properties of 8BrG lesions have not yet been examined, we believe that our present findings, which indicated that G → T, G → C, G → A, and delG mutations were induced by 8BrG in human cells, are compatible with the results of these two previous reports. Additionally, the presence of both the most predominant mutation type (i.e., G → T mutation) and the second most predominant mutation type (i.e., G → C mutation) induced by 8BrG in three different cell lines seems to strengthen our conclusion regarding 8BrG-induced mutation types.

Regarding 8BrG-induced G → T mutations, our knowledge of the DNA polymerase responsible for this translesion synthesis is limited. According to previous *in vitro* analyses, Pol*κ*, Pol*α*, and Pol*β* are involved in the misincorporation of A opposite 8BrG at a low frequency [13, 14], and the involvement of such misincorporation has been speculated to occur with other translesion synthesis polymerases. In another point of view, both 8BrG and 8oxoG are C8-modified guanines, and 8oxoG in its *syn* conformation can form two H-bonds with *anti*-A using an H-bond donor and an H-bond acceptor on its Hoogsteen edge, causing G → T mutation [30]. Similarly, 8BrG in its *syn* conformation has been speculated to form two H-bonds with *anti*-A [14], although a direct X-ray structure analysis is not yet available to substantiate this point. Future analyses of the translesion synthesis and X-ray structure of 8BrG should help to clarify the mechanism of 8BrG-induced G → T mutations.

According to previous *supF* forward mutation assays using wild-type pMY189 in human cells [31, 32], a G:C to T:A mutation was the most predominant mutation type among base substitution mutations at any G positions in the *supF* gene on untreated pMY189, although G:C to C:G and G:C to A:T mutations were also detected at lower frequencies (Supplementary Table S3). This mutation spectrum resembles the mutation spectrum at position 159, where 8BrG was introduced, in our study. However, the frequency of mutant colonies containing a base substitution at position 159 of *supF* on untreated pMY189 was extremely low in the two previous studies (0% and 0.3%) and in our study (1.1%). Thus, the mutation spectrum at position 159, an 8BrG-introduced site, in our study can likely be largely ascribed to 8BrG.

Yasui et al. [33] recently investigated the prevalence of mutations at 8BrG-introduced sites in the human genome using a system for tracing DNA adducts in targeted mutagenesis, which is different from a *supF* forward mutation assay. They showed that the frequency of targeted mutants arising from the introduction of 8BrG was 0.4%, while the frequency of the wild-type sequence was 0.1%, in Supplementary Material section of their paper. On the other hand, our present *supF* forward mutation assay performed in H1299 cells showed that the frequency of targeted mutants arising from the introduction of 8BrG was 0.06% [0.272 × 0.224; (mutation frequency) × (percentage of mutant colonies containing a mutation at 8BrG-introduced site of *supF*)] and that of the wild-type sequence was 0.0007% (0.06 × 0.011). The difference in frequencies between their study and ours is likely the result of differences in the assay systems and cell lines that were used. However, more importantly, the results of their study and ours indicate that 8BrG is indeed mutagenic. Thus, 8BrG is likely to be involved in human diseases, including cancer, through their ability to induce mutation.

In our *supF* forward mutation assay using a pMY189 plasmid containing an 8BrG at position 159 of *supF*, the frequency of mutant colonies not containing a point mutation at position 159 ranged from 75.7% to 79.7% in the three human cell lines that were used (Figure 1(b)). The mutations seen in these mutant colonies were off-target mutations composed of point mutations at a position other than 159 or large insertions/deletions. At present, determining whether these off-target mutations can be ascribed to 8BrG is difficult, since the mutagenicity of 8BrG at sites other than 8BrG-introduced sites is poorly understood.

The excisional activity of MUTYH towards A mispaired with 8BrG was clearly shown in our DNA cleavage activity assay. This activity is compatible with a previous observation of a direct connection between the *E. coli* DNA glycosylase MutY, a homologue of MUTYH, and 8BrG-containing oligonucleotides in a UV cross-linking analysis [34]. However, since whether human MUTYH protein possesses repair activity towards 8BrG has not yet been reported, our study is the first to demonstrate the excisional activity of MUTYH towards A:8BrG. Additionally, we showed that MUTYH possesses the ability to suppress G → T mutations caused by 8BrG in a *supF* forward mutation assay using *MUTYH*-overexpressing human cells and control cells. *MUTYH* is the responsible gene for MUTYH-associated polyposis (MAP), a hereditary disease characterized by colorectal polyposis and carcinoma(s) [10, 17]. Somatic G → T mutations in the *APC* and *KRAS* genes are frequently observed in such colorectal tumors in MAP patients [10, 17], and even in non-MAP patients, reduced MUTYH expression is associated with an increased number of somatic G → T mutations in prostate adenocarcinoma [35]. So far, a decrease in the repair activity of MUTYH towards A:8oxoG has been considered a mechanism underlying the increase in the frequency of G → T mutations [10, 17]. Our results suggest that a decrease in the repair activity of MUTYH towards not only A:8oxoG but also A:8BrG could be associated with the above-described diseases, via the induction of G → T mutations.

Our DNA cleavage activity assay, which included a time-course assay and an assay using various amounts of DNA glycosylase proteins (Figures 2 and 3), clearly demonstrated the excisional activity of both SMUG1 and TDG towards T mispaired with 8BrG. Furthermore, based on these results, the repair activity towards the substrate seemed to be stronger for TDG than SMUG1 (average % incision at a 120 min reaction time: 88.5% for TDG and 14.6% for SMUG1). Calculation of the catalytic parameter also showed that the catalytic efficiency (*K*cat/*K*m) of the TDG protein was higher than that of the SMUG1 protein. SMUG1 has the ability to excise U, 5-hydroxyuracil, 5-hydroxymethyluracil (5hmU), 5-formyluracil, and 3,*N*^4^-ethenocytosine (*ε*C) [36, 37]; however, an ability to excise unmodified T opposite any damaged base has not been reported for SMUG1. On the other hand, TDG is known to be involved in the removal of T and U mispaired with G, as well as 5-fluorouracil, 5hmU, *ε*C, 5-methylcytosine, 5-formylcytosine, and 5-carboxycytosine bases [19, 38]. Recently, TDG was also shown to be involved in T mispairing with several types of exocyclic etheno-base lesions, such as *ε*C [29]. Thus, the identification of a novel substrate (i.e., T:8BrG) of SMUG1 and TDG proteins in the present study, in addition to the other known substrates described above, suggests that both SMUG1 and TDG exhibit a broad substrate specificity. Although the precise process responsible for the SMUG1-/TDG-initiated repair of T:8BrG mispairing is unclear at present, we suspected that the DNA glycosylase activities of both proteins towards T opposite 8BrG would have some effect on 8BrG-induced mutations in human cells.

The 5′- and 3′-flanking bases of the mispair site repaired by DNA glycosylase protein can reportedly affect the efficiency of the DNA glycosylase reaction [39]. The DNA substrates used in our study were 5′-C[8BrG]C-3′ (opposite base of 8BrG is T, C, G, or A) and 5′-G[T, C, G, or A]G-3′ (opposite base of central base is 8BrG), as shown in Supplementary Figure S3. We did not investigate the effects of substitutions at the 5′- and 3′-flanking bases on DNA cleavage. At present, we only know that A paired with 8BrG in an oligonucleotide was recognized and excised by MUTYH and that T paired with 8BrG in an oligonucleotide was recognized and excised by both SMUG1 and TDG. Future investigations of the 5′- and 3′-flanking bases would clarify the roles of MUTYH on the A:8BrG substrate and of SMUG1/TDG on the T:8BrG substrate in greater detail.

Among the 8BrG-induced mutation types found in our current analysis, the G → T mutation was the most frequent. The G → T (G:C to T:A) mutation is a predominant mutation type in both hepatitis B virus-positive and hepatitis C virus-positive hepatocellular carcinomas, which are both inflammation-related cancers [40]. Moreover, the G → T mutation is the most frequent or the second most frequent mutation type in many types of human cancers [41]. Since chronic inflammation is involved in the initiation of carcinogenesis and intratumoral inflammation accelerates cancer progression [1], it seems plausible that 8BrG-induced mutations might influence various types of cancers.

## Supplementary Material


**Supplementary Table S1:** Number of mutant clones analyzed in the PCR and gel electrophoresis and in the sequencing in the *supF* forward mutation assay. **Supplementary Table S2:** Primers used for PCR amplification of *TDG* and *SMUG1* coding exons. **Supplementary Table S3:** Mutation spectrum of base substitution mutations at the G position in the *supF* gene on the shuttle vector plasmid pMY189 replicated in human cells, based on previous reports. **Supplementary Figure S1:** Proportion of mutation types detected at position 144 of *supF* on 8BrG-containing pMY189 plasmids, which has an 8BrG residue at position 144 of *supF*, replicated in H1299 cells. **Supplementary Figure S2:** Detection of the DNA glycosylase activity of each DNA glycosylase protein on double-stranded oligonucleotides containing the previously reported substrate using a DNA cleavage activity assay. **Supplementary Figure S3:** Substrates used in the DNA cleavage activity assay. **Supplementary Figure S4:** Comparison of excisional activities of TDG proteins between thymine mispaired with 8-bromoguanine and thymine mispaired guanine. **Supplementary Figure S5:** Excision statuses of SMUG1 and TDG proteins for T paired with 8BrG, G, C, or A. **Supplementary Figure S6:** TDG and SMUG1 protein expression levels in LN428 cells.

## Figures and Tables

**Table 1:** Kinetic constants of the TDG and SMUG1 proteins for the excision of thymine mispaired with 8BrG.

Type of protein	*K*m (nM)	*K*cat (min^−1^)	*K*cat/*K*m (min^−1^·*μ*M^−1^)
TDG	0.72	0.042	58.5
SMUG1	2.63	0.011	4.2

## References

[1] Kundu J. K., Surh Y. J. (2012). Emerging avenues linking inflammation and cancer. *Free Radical Biology and Medicine*.

[2] Tafani M., Sansone L., Limana F. (2016). The interplay of reactive oxygen species, hypoxia, inflammation, and sirtuins in cancer initiation and progression. *Oxidative Medicine and Cellular Longevity*.

[3] Thomas E. L., Bozeman P. M., Jefferson M. M., King C. C. (1995). Oxidation of bromide by the human leukocyte enzymes myeloperoxidase and eosinophil peroxidase. Formation of bromamines. *Journal of Biological Chemistry*.

[4] Gaut J. P., Yeh G. C., Tran H. D. (2001). Neutrophils employ the myeloperoxidase system to generate antimicrobial brominating and chlorinating oxidants during sepsis. *Proceedings of the National Academy of Sciences of the United States of America*.

[5] Shen Z., Mitra S. N., Wu W. (2001). Eosinophil peroxidase catalyzes bromination of free nucleosides and double-stranded DNA. *Biochemistry*.

[6] Pattison D. I., Davies M. J. (2004). Kinetic analysis of the reactions of hypobromous acid with protein components: implications for cellular damage and use of 3-bromotyrosine as a marker of oxidative stress. *Biochemistry*.

[7] Asahi T., Kondo H., Masuda M. (2010). Chemical and immunochemical detection of 8-halogenated deoxyguanosines at early stage inflammation. *Journal of Biological Chemistry*.

[8] Domingueti C. P., Dusse L. M., Carvalho M. d., de Sousa L. P., Gomes K. B., Fernandes A. P. (2016). Diabetes mellitus: the linkage between oxidative stress, inflammation, hypercoagulability and vascular complications. *Journal of Diabetes and its Complications*.

[9] Cheng K. C., Cahill D. S., Kasai H., Nishimura S., Loeb L. A. (1992). 8-Hydroxyguanine, an abundant form of oxidative DNA damage, causes G → T and A → C substitutions. *Journal of Biological Chemistry*.

[10] Suzuki T., Kamiya H. (2016). Mutations induced by 8-hydroxyguanine (8-oxo-7,8-dihydroguanine), a representative oxidized base, in mammalian cells. *Genes and Environment*.

[11] Cooke M. S., Evans M. D., Dizdaroglu M., Lunec J. (2003). Oxidative DNA damage: mechanisms, mutation, and disease. *FASEB Journal*.

[12] Jena N. R., Mishra P. C. (2012). Formation of ring-opened and rearranged products of guanine: mechanisms and biological significance. *Free Radical Biology and Medicine*.

[13] Sassa A., Ohta T., Nohmi T., Honma M., Yasui M. (2011). Mutational specificities of brominated DNA adducts catalyzed by human DNA polymerases. *Journal of Molecular Biology*.

[14] Efrati E., Tocco G., Eritja R., Wilson S. H., Goodman M. F. (1999). “Action-at-a-distance” mutagenesis. 8-Oxo-7,8-dihydro-2′-deoxyguanosine causes base substitution errors at neighboring template sites when copied by DNA polymerase β. *Journal of Biological Chemistry*.

[15] Lange S. S., Takata K., Wood R. D. (2011). DNA polymerases and cancer. *Nature Reviews Cancer*.

[16] Tsuzuki T., Nakatsu Y., Nakabeppu Y. (2007). Significance of error-avoiding mechanisms for oxidative DNA damage in carcinogenesis. *Cancer Science*.

[17] Shinmura K., Goto M., Tao H., Sugimura H. (2012). Role of base excision repair enzyme MUTYH in the repair of 8-hydroxyguanine and MUTYH-associated polyposis (MAP). *Hereditary Genetics*.

[18] Krokan H. E., Bjørås M. (2013). Base excision repair. *Cold Spring Harbor Perspectives in Biology*.

[19] Cortázar D., Kunz C., Saito Y., Steinacher R., Schär P. (2007). The enigmatic thymine DNA glycosylase. *DNA Repair*.

[20] Marnett L. J., Plastaras J. P. (2001). Endogenous DNA damage and mutation. *Trends in Genetics*.

[21] Jena N. R. (2012). DNA damage by reactive species: mechanisms, mutation and repair. *Journal of Biosciences*.

[22] Cozens A. L., Yezzi M. J., Kunzelmann K. (1994). CFTR expression and chloride secretion in polarized immortal human bronchial epithelial cells. *American Journal of Respiratory Cell and Molecular Biology*.

[23] Matsuda T., Yagi T., Kawanishi M., Matsui S., Takebe H. (1995). Molecular analysis of mutations induced by 2-chloroacetaldehyde, the ultimate carcinogenic form of vinyl chloride, in human cells using shuttle vectors. *Carcinogenesis*.

[24] Kawanishi M., Matsuda T., Sasaki G., Yagi T., Matsui S., Takebe H. (1998). A spectrum of mutations induced by crotonaldehyde in shuttle vector plasmids propagated in human cells. *Carcinogenesis*.

[25] Akasaka S., Takimoto K., Yamamoto K. (1992). G:C → T:A and G:C → C:G transversions are the predominant spontaneous mutations in the *Escherichia coli supF* gene: an improved *lacZ(am) E. coli* host designed for assaying pZ189 *supF* mutational specificity. *Molecular and General Genetics*.

[26] Shinmura K., Goto M., Suzuki M. (2011). Reduced expression of MUTYH with suppressive activity against mutations caused by 8-hydroxyguanine is a novel predictor of a poor prognosis in human gastric cancer. *Journal of Pathology*.

[27] Shinmura K., Tao H., Goto M. (2004). Inactivating mutations of the human base excision repair gene *NEIL1* in gastric cancer. *Carcinogenesis*.

[28] Goto M., Shinmura K., Nakabeppu Y. (2010). Adenine DNA glycosylase activity of 14 human MutY homolog (MUTYH) variant proteins found in patients with colorectal polyposis and cancer. *Human Mutation*.

[29] Goto M., Shinmura K., Matsushima Y. (2014). Human DNA glycosylase enzyme TDG repairs thymine mispaired with exocyclic etheno-DNA adducts. *Free Radical Biology and Medicine*.

[30] Batra V. K., Beard W. A., Hou E. W., Pedersen L. C., Prasad R., Wilson S. H. (2010). Mutagenic conformation of 8-oxo-7,8-dihydro-2′-dGTP in the confines of a DNA polymerase active site. *Nature Structural & Molecular Biology*.

[31] Kawanishi M., Matsuda T., Nakayama A., Takebe H., Matsui S., Yagi T. (1998). Molecular analysis of mutations induced by acrolein in human fibroblast cells using *supF* shuttle vector plasmids. *Mutation Research*.

[32] Sunaga N., Kohno T., Shinmura K. (2001). OGG1 protein suppresses G:C→T:A mutation in a shuttle vector containing 8-hydroxyguanine in human cells. *Carcinogenesis*.

[33] Yasui M., Kanemaru Y., Kamoshita N., Suzuki T., Arakawa T., Honma M. (2014). Tracing the fates of site-specifically introduced DNA adducts in the human genome. *DNA Repair*.

[34] Bernards A. S., Miller J. K., Bao K. K., Wong I. (2002). Flipping duplex DNA inside out: a double base-flipping reaction mechanism by *Escherichia coli* MutY adenine glycosylase. *Journal of Biological Chemistry*.

[35] Shinmura K., Kato H., Kawanishi Y. (2017). Reduced expression of the DNA glycosylase gene MUTYH is associated with an increased number of somatic mutations via a reduction in the DNA repair capacity in prostate adenocarcinoma. *Molecular Carcinogenesis*.

[36] Kavli B., Sundheim O., Akbari M. (2002). hUNG2 is the major repair enzyme for removal of uracil from U:A matches, U:G mismatches, and U in single-stranded DNA, with hSMUG1 as a broad specificity backup. *Journal of Biological Chemistry*.

[37] Masaoka A., Matsubara M., Hasegawa R. (2003). Mammalian 5-formyluracil-DNA glycosylase. 2. Role of SMUG1 uracil-DNA glycosylase in repair of 5-formyluracil and other oxidized and deaminated base lesions. *Biochemistry*.

[38] Maiti A., Drohat A. C. (2011). Thymine DNA glycosylase can rapidly excise 5-formylcytosine and 5-carboxylcytosine: potential implications for active demethylation of CpG sites. *Journal of Biological Chemistry*.

[39] Hang B., Sági J., Singer B. (1998). Correlation between sequence-dependent glycosylase repair and the thermal stability of oligonucleotide duplexes containing 1,*N*^6^-ethenoadenine. *Journal of Biological Chemistry*.

[40] Fujimoto A., Totoki Y., Abe T. (2012). Whole-genome sequencing of liver cancers identifies etiological influences on mutation patterns and recurrent mutations in chromatin regulators. *Nature Genetics*.

[41] Kandoth C., McLellan M. D., Vandi F. (2013). Mutational landscape and significance across 12 major cancer types. *Nature*.

