# Supplementary material for: Mutation Spectrum Induced by 8-Bromoguanine, a Base Damaged by Reactive Brominating Species, in Human Cells
Source: Oxid Med Cell Longev. 2017 Sep 30;2017:7308501. doi: 10.1155/2017/7308501 (PMC5643121; doi:10.1155/2017/7308501)
Supplement: Supplementary file 1 — Supplementary Table S1: Number of mutant clones analyzed in the PCR and gel electrophoresis and in the sequencing in the supF forward mutation assay. Supplementary Table S2: Primers used for PCR amplification of TDG and SMUG1 coding exons. Supplementary Table S3: Mutation spectrum of base substitution mutations at the G position in the supF gene on the shuttle vector plasmid pMY189 replicated in human cells, based on previous reports. Supplementary Figure S1: Proportion of mutation types detected at position 144 of supF on 8BrG-containing pMY189 plasmids, which has an 8BrG residue at position 144 of supF, replicated in H1299 cells. Supplementary Figure S2: Detection of the DNA glycosylase activity of each DNA glycosylase protein on double-stranded oligonucleotides containing the previously reported substrate using a DNA cleavage activity assay. Supplementary Figure S3: Substrates used in the DNA cleavage activity assay. Supplementary Figure S4: Comparison of excisional activities of TDG proteins between thymine mispaired with 8-bromoguanine and thymine mispaired guanine. Supplementary Figure S5: Excision statuses of SMUG1 and TDG proteins for T paired with 8BrG, G, C, or A. Supplementary Figure S6: TDG and SMUG1 protein expression levels in LN428 cells. [file 7308501.f1.docx]

**Supplementary Information**

**Mutation Spectrum Induced by 8-Bromoguanine, a Base Damaged by Reactive Brominating Species, in Human Cells**

**Shinmura K *et al.***

**Submitted to *Oxidative Medicine and Cellular Longevity***

**Supplementary Table S1:** Number of mutant clones analyzed in the PCR and gel electrophoresis and in the sequencing in the *supF* forward mutation assay.

**Supplementary Table S2:** Primers used for PCR amplification of *TDG* and *SMUG1* coding exons.

**Supplementary Table S3:** Mutation spectrum of base substitution mutations at the G position in the *supF* gene on the shuttle vector plasmid pMY189 replicated in human cells, based on previous reports.

**Supplementary Figure S1:** Proportion of mutation types detected at position 144 of *supF* on 8BrG-containing pMY189 plasmids, which has an 8BrG residue at position 144 of *supF,* replicated in H1299 cells.

**Supplementary Figure S2:** Detection of the DNA glycosylase activity of each DNA glycosylase protein on double-stranded oligonucleotides containing the previously reported substrate using a DNA cleavage activity assay.

**Supplementary Figure S3:** Substrates used in the DNA cleavage activity assay.

**Supplementary Figure S4:**  Comparison of excisional activities of TDG proteins between thymine mispaired with 8-bromoguanine and thymine mispaired guanine.

**Supplementary Figure S5:**  Excision statuses of SMUG1 and TDG proteins for T paired with 8BrG, G, C, or A.

**Supplementary Figure S6:**  TDG and SMUG1 protein expression levels in LN428 cells.

**Supplementary Table S1.** Number of mutant clones analyzed in the PCR and gel electrophoresis and in the sequencing in the *supF* forward mutation assay

| Cell line |  | Plasmid (pMY189) | Number of mutant clones analyzed^a^ | |
| --- | --- | --- | --- | --- |
|  |  |  | PCR and gel electrophoresis | Sequencing |
| H1299 | Human lung cancer cell line | Wild-type pMY189 | 90 | 25 |
| H1299 | Human lung cancer cell line | 8BrG-containing pMY189 | 331 | 158 |
| LN428 | Human glioblastoma cell line | 8BrG-containing pMY189 | 232 | 190 |
| 16HBE14o- | Simian virus 40-transformed human bronchial epithelial cell line | 8BrG-containing pMY189 | 74 | 49 |
| MUTYH-overexpressing H1299 cells (cumate-inducible cells) | | 8BrG-containing pMY189 | 82 | 46 |
| Empty vector (PiggyBac)  -transposed H1299 cells | | 8BrG-containing pMY189 | 115 | 55 |

^a^PCR products covering the *supF* gene were analyzed by agarose gel electrophoresis at first. If the PCR product of mutant colonies showed apparently the same mobility as that of wild-type clone, the PCR product was then analyzed by sequencing.

**Supplementary Table S2.** Primers used for PCR amplification of *TDG* and *SMUG1* coding exons

| Gene | Exon | Forward sequence (5'→3') | Reverse sequence (5'→3') | size (bp) |
| --- | --- | --- | --- | --- |
| *TDG* | 1 | GCTTGAGTCCAGCCACTGTCT | ATAGTCAACTGGTTATCGCGGG | 426 |
| *TDG* | 2 | ACAGTGCAAGAGTTAATGGTTGGTT | ACCCATGTTCTTTTATTCCCCAG | 535 |
| *TDG* | 3 | AGACTCATGTTGGGACTGTAAGAGC | CTCATGAAGCTGACACCATTTACC | 522 |
| *TDG* | 4 | GGGATCATAAAGGAGTCAAACTGG | GACACACACACAGAACATGAAACAC | 457 |
| *TDG* | 5 | AAAATTAGCTGGGCGTGGTG | CGTCCTCCTTCACGAAATTCTT | 548 |
| *TDG* | 6&7 | CCCCAAATATTCTAGGAACATCATATGT | ACATCAAGACATTTAGCTGACAAATGTT | 628 |
| *TDG* | 8 | CAACCTTAAAAGCAACTACTTTTATTGA | TACACACACAAAATGAATAAAAGGAAT | 372 |
| *TDG* | 9 | CCTACGTAAAGTTGAATGTAAGCGG | AAGTCTGTGTAATTCAACGTGAAATAACC | 501 |
| *TDG* | 10 | AGGATCACTCAAGCCCAGGA | CTACTTAGGTTCATACGGTTCACACTACC | 462 |
| *SMUG1* | 3 | AGGAGCCAGTATATGTGTTGAGACAG | GGTTGGTAGATGACTGACATGGTC | 588 |
| *SMUG1* | 4-1 | TCAGGAGGGCAAACAGGAGTA | GCCCACAGTCAGAAGTGAGTG | 580 |
| *SMUG1* | 4-2 | CCTTGTTGGCCTGTGGGTTA | CAGGCCTTTGGGTGAAGGTA | 598 |

**Supplementary Table S3.** Mutation spectrum of base substitution mutations at the G position in the *supF* gene on the shuttle vector plasmid pMY189 replicated in human cells, based on previous reports

| Cell | Mutation type | | | Frequency of mutant colonies containing a base substitution at position 159 of *supF* | Reference |
| --- | --- | --- | --- | --- | --- |
|  | G:C to T:A | G:C to C:G | G:C to A:T |  |  |
| WI38-VA13 | 56% | 21% | 21% | 0% | Kawanishi et al., 1998 |
| H1299 | 52% | 28% | 20% | 0.3% | Sunaga  et al., 2001 |

Reference

M. Kawanishi, T. Matsuda, A. Nakayama, H. Takebe, S. Matsui, and T. Yagi, “Molecular analysis of mutations induced by acrolein in human fibroblast cells using supF shuttle vector plasmids,” *Mutation Research*, vol. 417, no. 2-3, pp. 65-73, 1998.

N. Sunaga, T. Kohno, K. Shinmura et al., “OGG1 protein suppresses G:C-->T:A mutation in a shuttle vector containing 8-hydroxyguanine in human cells,” *Carcinogenesis*, vol. 22, no. 9, pp. 1355-1362, 2001.

**Supplementary Figure S1.** Proportion of mutation types detected at position 144 of *supF* on 8BrG-containing pMY189 plasmids, which has an 8BrG residue at position 144 of *supF,* replicated in H1299 cells.

**Supplementary Figure S2.** Detection of the DNA glycosylase activity of each DNA glycosylase protein on double-stranded oligonucleotides containing the previously reported substrate using a DNA cleavage activity assay. (A) The DNA glycosylase proteins MUTYH, MPG, NEIL1, OGG1, SMUG1, TDG, UNG2, and NTHL1 was allowed to act on ^32^P-labeled double-stranded oligonucleotide containing each mismatch at 37°C for 60 min, and reaction mixture was subjected to 20% PAGE. HI means heat-inactivation, which was performed by heating the protein at 100°C for 5 min before the DNA cleavage activity assay. An asterisk indicates a ^32^P-labeled oligonucleotide. The intact oligonucleotides and cleavage products are indicated by “I” and “C”, respectively. (B) Information of double-stranded oligonucleotide used in the experiments in (A) was summarized. εA, Tg, 8oxoG, and U mean 1,N^6^-ethenoadenine, thymine glycol, 8-oxoguanine, and uracil, respectively.

**Supplementary Figure S3.** Substrates used in the DNA cleavage activity assay. A total of eight kinds of ^32^P-labeled 30-mer double-stranded oligonucleotides were prepared. The asterisks show the 5'-^32^P-labeled oligonucleotides.

**Supplementary Figure S4.** Comparison of excisional activities of TDG proteins between thymine mispaired with 8-bromoguanine (8BrG) and thymine mispaired with guanine. Time-course assay for the cleavage of 30-mer double-stranded oligonucleotides containing a T:8BrG mispair or a T:G mispair by TDG proteins was performed. Each protein or no DNA glycosylase proteins (-) was incubated at 37°C for 0-120 min with a T:8BrG- or T:G-containing oligonucleotide. The amount of cleavage products as a proportion of the total oligonucleotides was calculated as the % incision. The % incision values were shown as the means ± standard deviations of data from three independent experiments. The lower panels show representative results of the DNA cleavage activity assays. The asterisks show the 5'-^32^P-labeled oligonucleotides. A ^32^P-labeled marker oligonucleotide was used as a size marker for the cleavage products. The intact oligonucleotides and cleavage products are indicated by “I” and “C”, respectively.

**Supplementary Figure S5.** Excision statuses of SMUG1 and TDG proteins for T paired with 8BrG, G, C, or A. Each DNA glycosylase protein was allowed to act on double-stranded oligonucleotides containing T paired with 8BrG, G, C, or A at 37°C for 60 min. The asterisks show the 5'-^32^P-labeled oligonucleotides. A ^32^P-labeled marker oligonucleotide was used as a size marker for the cleavage products. The intact oligonucleotides and cleavage products are indicated by “I” and “C”, respectively.

**Supplementary Figure S6.** TDG and SMUG1 protein expression levels in LN428 cells. Cultured cells were lysed in a buffer containing 50 mM HEPES-KOH (pH7.5), 150 mM NaCl, 0.1% sodium dodecyl sulfate, 1% Triton X-100, 0.5% sodium deoxycholate, 100 mM sodium fluoride, 1 mM sodium orthovanadate, and protease inhibitor cocktail (Sigma, St Louis, MO, USA). A Western blot analysis was performed using an anti-TDG polyclonal antibody (Bioworld Technology, Minneapolis, MN, USA), an anti-SMUG1 polyclonal antibody (Sigma-Aldrich, St. Louis, MO, USA), or an anti-GAPDH monoclonal antibody (clone 6C5; Abcam, Cambridge, UK). Immunoreactivity was visualized using an ECL chemiluminescence system (GE Healthcare Bio-Science, Piscataway, NJ, USA). ImageJ software (National Institutes of Health, Bethesda, MD, USA) was used to measure the protein expression level. The values of (TDG expression level)/(GAPDH expression level) and (SMUG1 expression level)/(GAPDH expression level) in 16HBE14o- cells were set equal to 1.0.
